# Deciphering and advancing CAR T-cell therapy with single-cell sequencing technologies

**DOI:** 10.1186/s12943-023-01783-1

**Published:** 2023-05-06

**Authors:** Shengkang Huang, Xinyu Wang, Yu Wang, Yajing Wang, Chenglong Fang, Yazhuo Wang, Sifei Chen, Runkai Chen, Tao Lei, Yuchen Zhang, Xinjie Xu, Yuhua Li

**Affiliations:** 1 The Second School of Clinical Medicine, Zhujiang Hospital, Southern Medical University, Guangzhou, China; 2 State Key Laboratory of Cardiovascular Disease, Fuwai Hospital, National Center for Cardiovascular Diseases, Chinese Academy of Medical Sciences and Peking Union Medical College, Beijing, China; 3 The First School of Clinical Medicine, Nanfang Hospital, Southern Medical University, Guangzhou, China; 4 School of Rehabilitation Sciences, Southern Medical University, Guangzhou, China; 5 Department of Hematology, Zhujiang Hospital, Southern Medical University, Guangzhou, China; 6 Bioland Laboratory (Guangzhou Regenerative Medicine and Health Guangdong Laboratory), Guangzhou, China

**Keywords:** CAR T-cell, Single-cell sequencing technologies, Biology, Mechanisms, Strategies, Target selection

## Abstract

Chimeric antigen receptor (CAR) T-cell therapy has made remarkable progress in cancer immunotherapy, but several challenges with unclear mechanisms hinder its wide clinical application. Single-cell sequencing technologies, with the powerful unbiased analysis of cellular heterogeneity and molecular patterns at unprecedented resolution, have greatly advanced our understanding of immunology and oncology. In this review, we summarize the recent applications of single-cell sequencing technologies in CAR T-cell therapy, including the biological characteristics, the latest mechanisms of clinical response and adverse events, promising strategies that contribute to the development of CAR T-cell therapy and CAR target selection. Generally, we propose a multi-omics research mode to guide potential future research on CAR T-cell therapy.

## Introduction

Chimeric antigen receptor (CAR) T-cell therapy has changed the therapeutic landscape of cancer immunotherapy, especially for hematological malignancies, including B-cell acute lymphoblastic leukemia (B-ALL) [1], B-cell non-Hodgkin’s lymphoma (B-NHL) [2], chronic lymphocytic leukemia (CLL) [3], and Hodgkin’s lymphoma (HL) [4]. Although CAR T-cell therapy has impressive clinical outcomes, there are still a series of challenges, such as high cost, restricted clinical accessibility, resistance, relapse and adverse events [5, 6]. The widespread application of CAR T-cell therapy in hematological malignancies has led researchers to test its efficacy in solid tumors. [7–11]. However, compared with hematological diseases, CAR T-cells are less effective in solid tumors due to the inadequate tumor infiltration of CAR T-cells, the lack of stably expressed, tumor-specific antigens, the highly immunosuppressive tumor microenvironment (TME), etc. [12–16]. These problems are expected to be addressed by analyzing the molecular landscape and heterogeneity of CAR T-cells and their interactions with various cells in the microenvironment.

Flow cytometry has been the main metric used in CAR T-cell therapy to measure the immunophenotype and composition of circulating CAR T-cells [17, 18], but its low throughput and hypothesis-driven nature prevent unbiased exploratory screening and molecular profiling of CAR T-cells. Therefore, it is challenging to identify the key molecular drivers associated with the expansion and persistence properties of CAR T-cells that are directly related to treatment outcomes. These shortcomings in measuring clinically relevant features are being addressed with the development of single-cell RNA sequencing (scRNA-seq). Single-cell sequencing technologies enable unbiased, high-resolution, and high-throughput sequencing analysis to reveal cellular heterogeneity with unprecedented resolution and capture high levels of the molecular landscape. Functional analysis also helps to understand cellular transitions, interactions and communications of CAR T-cells [19–23]. This has provided new insights into the overall performance of CAR T-cells in relation to patient prognosis (Table 1). In this review, we discuss how single-cell sequencing technologies, especially scRNA-seq, decipher and advance CAR T-cells and facilitate CAR target selection. Finally, we propose a multi-omics research mode from a clinically translational perspective to help researchers better understand and explore the future directions of CAR T-cell therapy.

**Table 1:** Selected published articles of CAR T-cell therapy with single-cell sequencing technologies

Technology	Tumor type	Target antigen	Sample	Sample source	Research level	Cell number	Experimental design	Conclusion	Citation
CAR T-cell product heterogeneity									
scRNA-seq, bulk RNA-seq	B-ALL	CD19	Activated and inactivated CAR T-cells	5 healthy donors	Cell	83,123	Bulk RNA-seq and scRNA-seq were performed on antigen-specific stimulated and unstimulated CAR T-cells with non-costimulatory domains and different costimulatory domains (CD28,4-1BB).	Compared to CD28 CAR T-cells, 4-1BB CAR T-cells enriched in a central memory cell phenotype and fatty acid metabolism genes. And 4-1BB CAR T-cells also had increased expression of MHC II genes, ENPP2, and IL-21 axis genes, and decreased PD1.	[59]
Bulk RNA-seq, CITE-seq, scATAC-Seq	B-ALL	CD19	Post-infusion CAR T-cells	71 patients	Human	37,423	RNA-seq was performed on a classified CAR T-cell subsets from 71 patients, followed by matching CITE-seq and scATAC-seq on T cells from 6 of these patients.	The TCF7 regulon was not only associated with the favorable naïve T-cell state, but maintained in effector T cells among patients with long-term CAR T-cell persistence; chronic IFN signaling regulated by IRF7 was associated with poor CAR T-cell persistence across T cell subsets.	[34]
scRNA-seq, CITE-seq	ALL	CD19	Activated and inactivated CAR T-cells	1 healthy donor and 2 patients	Cell	23,349	scRNA-seq and CITE-seq were performed on CAR T-cells that were stimulated specifically or non-specifically with cells expressing CD19 or MSLN respectively.	Delineate the global cellular and molecular CAR T-cell landscape at baseline or in the activated state. Besides, healthy donor-derived CAR T-cells manifestated stronger functional activities correlated with the upregulation of MHC II genes than patient-derived CAR T-cells.	[61]
scRNA-seq, ATAC-seq, flow cytometry	MM	BCMA	IPs	13 healthy donors, public bulk RNA-seq and scRNA-seq datasets	Human	43,981	Detect the antitumor response of CAR^High^ and CAR^Low^ T-cells in vitro and in vivo. RNA-seq and ATAC-seq were performed on CAR^High^ and CAR^Low^ T-cells and scRNA-seq was performed on IPs from 3 independent donors. Analyze the correlation between CAR^High^ T-cell gene signature and clinical response through bulk RNA-seq and scRNA-seq datasets.	Reveal the different profiles between CAR^High^ and CAR^Low^ T-cells in phenotypic, functional, transcriptomic and epigenomic levels and provide mechanistic insights behind differential functionality of these cells. Particularly, CAR^High^ T-cells were related to tonic signaling and a cell exhausted phenotype as well as an increase in tumor cytotoxicity in vitro. Patients treated with CAR T-cell products enriched in CAR^High^ T cells showed a significantly worse clinical response.	[71]
scRNA-seq, flow cytometry	ALL	CD19	Activated CAR T-cells	3 donors	Cell	31,000	Flow cytometry and scRNA-seq were performed on antigen-stimulated CAR T-cells that were not transduced and that were transduced by different lentiviral vectors (CD8-LV, VSV-LV).	VSV-LV CAR T-cells produced a more significant central memory phenotype, while CD8-LV CAR T-cells showed stronger cytotoxic activity.	[72]
**Antigen-specific stimulation of CAR T-cells**									
scRNA-seq, single-cell cytokine assay, single-cell cytotoxicity assay	BCL	CD19	Activated and inactivated CAR T-cells	3 healthy donors	Cell	3,817	scRNA-seq, single-cell cytokine assay, single-cell cytotoxicity assay were performed on antigen-specific stimulated or unstimulated CAR T-cells.	The activation states of CAR T-cells were highly mixed with T_H_1,T_H_2, Treg, and GM-CSF-expressing T cell responses in the same single cells and largely independent of differentiation status.	[75]
Flow cytometry, scRNA-seq	MM	BCMA, TACI	Activated and inactivated CAR T-cells	3 healthy donors	Cell	53,191	Combine flow cytometry and scRNA-seq to characterize three stages of the CAR T-cell production process, namely the starting leukapheresis sample, CAR-T-cell product, and the cellular product upon specific antigen stimulation.	CAR T-cell products from different donors showed a similar cellular composition, and only half of CAR-expressing cells displayed transcriptional changes upon CAR-specific antigen exposure. Particularly, a small proportion of antigen-responding CAR-expressing cells showed no transcriptional response to specific antigen stimulation and some stimulated CAR-expressing cells exhibited exhaustion features.	[74]
**Dynamic performance of CAR T-cells**									
scRNA-seq, scTCR-seq	B-ALL	CD19	IPs, post-infusion CAR T-cells	15 patients	Human	184,791	scRNA-seq and scTCR-seq were performed on IPs and PBMCs and BM-derived CAR T-cells at multiple time points after infusion (week 1–4/8, month 3/6).	TIGIT^+^CD27^−^CD62L^Low^ was identified and validated in the pre-infusion product cell subsets, resulting in a highly efficient post-infusion CAR T-cell phenotype.	[76]
TCR-seq, scRNA-seq	CLL, NHL	CD19	IPs, post-infusion CAR T-cells	10 patients	Human	62,167	TCR-seq was performed on CD8^+^ CAR T-cells before infusion and on day 7–14/26–30 after infusion, and scRNA-seq was performed on CD8^+^ CAR T- cells on day 7–14/26–30/83–112 after infusion .	Clonal diversity of CAR T-cells was highest in the IPs and declined following infusion. Clones expanding after infusion mainly originated from infused clusters with higher expression of cytotoxicity and proliferation genes.	[36]
CyTOF, scRNA-seq, CITE-seq, scTCR-seq	CLL	CD19	Post-infusion CAR T-cells	2 patients	Human	1,437	CyTOF was performed on CAR T-cells from 2 patients followed for 9.3 and 7.2 years, followed by scTCR-seq, CITE- seq, and scRNA-seq for late-stage CD4^+^ CAR T-cells.	There were two different phases of anti-leukemia response: an initial stage dominated by CD8^+^ and γδ CAR T-cells, followed by a long-term remission stage characterized by Ki67^hi^CD4^+^ CAR T-cells that exhibited proliferative and cytolytic phenotypes.	[81]
**Cellular interactions with CAR T-cells**									
scRNA-seq, flow cytometry	PCL	BCMA	IPs, post-infusion CAR T-cells	1 patient	Human	55,488	scRNA-seq was performed on CAR T -cells and endogenous T cells isolated from PBMC at three phases (day 0/8/15).	The mixed cell subsets mainly characterized by high metabolism of CD4^+^ T-cells in the initial stage would transition from highly amplified to cytotoxic CD8^+^ T-cells in the amplification stage. CD8^+^ memory-like cell states with high expression of RP genes were found in the cells on the final remission stage.	[80]
Image analysis, flow cytometry, scRNA-seq	BCL	CD19	BM cells	Mice	Mouse	NA	Flow cytometry and scRNA-seq were performed on BM cells from CAR T- cell-treated and untreated mice after 3 days of treatment.	CAR T-cells relied on cytokine-mediated crosstalk with the TME for optimal activity. IFN-γ produced by CAR T-cells enhanced endogenous T cells and sustained CAR T-cell cytotoxicity.	[85]
scRNA-seq, flow cytometry	GBM	IL13Rα2	CD45^+^ cells from the brains of untreated or CAR T-cell treated mice	Patients and mice	Human and Mouse	NA	Flow cytometry was performed on CAR T-cells after cell stimulation to measure IFN-γ production. scRNA-seq was performed on CD45^+^ cells from the brains of CAR T-cell treated or untreated mice.	IFN-γ production by CAR T-cells and IFN-γ responsiveness of host immune cells were critical for tumor immune landscape remodeling to promote a more activated and less suppressive tumor microenvironment.	[86]
scRNA-seq, bulk RNA-seq, ATAC-seq	PAAD	MSLN	Activated CAR T-cells, post-infusion CAR T-cells	Healthy donors and patients	Cell	16,000	Bulk RNA-seq and ATAC-seq were performed on M5CAR T-cells after CAE and T cell exhaustion related gene enrichment and scRNA-seq was performed on day 0/20/28 with CAE driver dysfunction.	CAE drove CAR T-cell exhaustion and promoted CD8^+^ CAR T-cell to NK-like CAR T-cell transition. ID3 and SOX4 were upregulated during the process of exhaustion and knocking out ID3 and SOX4 in CAR T-cells slowed dysfunction and improved antitumor immunity.	[87]
scATAC-seq, ChIP-seq, flow cytometry	B-ALL, Mm	CD19, BCMA	Activated CAR T-cells, post-infusion CAR T-cells	2 patients	Human	10,929	scATAC-seq was performed on CAR T-cells cultured in vitro and CAR T-cells of 2 Mm patients who received BCMA CAR T-cell therapy at the peak and decline of amplification.	Panoramic chromatin accessibility after CAR T- cell differentiation and exhaustion was depicted; BATF and IRF4 were key regulators of CAR T- cell exhaustion and downregulation of BATF and IRF4 contributed to reducing CAR T-cell exhaustion and enhancing CAR T-cell therapeutic efficacy.	[47]
**Primary resistance**									
scRNA-seq	LBCL	CD19	IPs	24 patients	Human	137,326	scRNA-seq was performed on IPs from 24 LBCL patients treated with CAR T- cells.	Within the IPs of patients with PR/PD, exhausted CD8^+^ and CD4^+^ T cells were significantly enriched, while memory type CD8 T cells were significantly enriched within the IPs of patients who achieved CR.	[88]
scRNA-seq, flow cytometry	NHL	CD19	IPs, post-infusion CAR T-cells	17 patients	Human	94,000	scRNA-seq and flow cytometry were performed on CAR T-cells from 17 NHL patients at different time points (IPs, day 14/30).	CD8^+^ CAR T-cells expressing exhausted marker TIGIT were associated with poor clinical response in NHL patients and targeted inhibition of TIGIT could improve the antitumor function of CAR T-cells.	[89]
scRNA-seq, TCR-seq, flow cytometry	BCL	CD19	Pre-infusion and post-infusion PBMCs, IPs	32 patients	Human	602,577	scRNA-seq and scTCR-seq performed on 105 pre-treatment and post-treatment PBMC samples at different time points (day 30 before treatment, day 7 after treatment ) and IPs collected from 32 individuals with BCL treated with axi-cel or tisa-cel.	Expansion of proliferative memory-like CD8 clones was a hallmark of tisa-cel response, whereas axi-cel responders displayed more heterogeneous populations. Besides, the higher number of CAR Treg cells was associated with disease progression.	[70]
scRNA-seq, genome-wide CRISPR/Cas9 knockout screening	B-ALL	CD19	IP, post-infusion CAR T-cells	2 patients	Human	NA	Genome-wide CRISPR/Cas9-based knockout screening was performed on the CD19^+^ human ALL cell line. scRNA-seq was performed on IPs from both responsive and unresponsive patients and on T cells at peak CAR T-cell amplification.	Death receptor signaling was identified as a key regulator of primary resistance to CAR T-cells in ALL. scRNA-seq confirmed that the CAR T-cells from patients with primary resistance expressed much higher levels of exhaustion markers.	[91]
**Relapse**									
CITE-seq, scRNA-seq, TCR-seq, flow cytometry	MM	BCMA	Pre-infusion and post-infusion BM cells	23 patients	Human	151,054	CITE-seq, scRNA-seq, TCR-seq, and flow cytometry were performed on BM samples from patients with long and short PFS before infusion and at day 28 and month 3 after infusion.	Short PFS was associated with the lower diversity of pretherapy TCR repertoire, presence of hyperexpanded clones with exhaustion phenotype, and BAFF^+^PD-L1^+^ myeloid cells in the marrow. And long PFS was associated with an increased proportion of CLEC9A^+^ DCs, CD27^+^TCF1^+^ T cells with diverse T-cell receptors, and emergence of T cells expressing marrow-residence genes.	[96]
scRNA-seq, scTCR-seq, cytokine multiplex profiling	MCL	CD19	Pre-infusion and post-infusion MCL cells and non-tumor cells of TME	20 patients	Human	40,091	scRNA-seq and scTCR-seq were performed on 39 samples collected over a long period of time from 15 CAR T-cell treated patients, and cytokine multiplex profiling was performed on 80 consecutive samples from 20 patients.	After relapse, CD4^+^ and CD8^+^ CTLs acquired expression of TIGIT and exhibited less cytotoxic. Besides, MCL tumor cells also increased TIGIT expression and then led to weaker antitumor immune surveillance. And elevated sIL2R in relapsed patients may contribute to therapeutic resistance by inhibiting T-cell expansion.	[98]
**Positive relapse**									
scRNA-seq, CITE-seq, flow cytometry, multiplexed secretomic assay	ALL	CD19	Activated and inactivated CAR T-cells, pre-infusion CAR T-cells	61 patients	Cell	101,326	scRNA-seq and CITE-seq were performed on (CAR-specific stimulation or TCR-mediated activation, not activated) IPs of 12 pediatric r/r ALL patients. Flow cytometry and multiplexed secretomic assay were performed on the pre-infusion CAR T-cells from other 49 patients.	The lack of T_H_2 function in CAR T-cell products was a novel mechanism for CD19-positive relapse and early memory-like T-cell subsets T_SCM_ and T_CM_ were significantly reduced in positive relapse patients.	[73]
**Negative relapse**									
scRNA-seq	B-ALL	CD19	Leukemia cells	1patient	Human	NA	scRNA-seq was performed on leukemic cells form BM before and after the patient received CD19 CAR-T cell therapy.	CD19-negative leukemic cells were present before CAR T-cell therapy and the relapse resulted from the selection of these rare pre-existing CD19-negative subclones.	[101]
scRNA-seq	B-ALL	CD19	CAR T-cells, leukemic cells	*	Cell	1,039	scRNA-seq was performed on the surviving leukemic cells cocultured with different methods (CAR T-cells and T cells) for 24 h.	Existing CD19^low^ leukemic cells sustained decreased CD19 expression through transcriptional programs of physiologic B-cell activation and germinal center reaction, which helped achieve immune escape.	[102]
Bulk RNA-seq, scRNA-seq, flow cytometry	B-ALL	CD19	HSPCs	2 second-trimester fetus	Human	30,000	Bulk RNA-seq, scRNA-seq and flow cytometry were performed on specific HSPC populations from FBM. CD34^+^CD19^−^CD22^+^ cells were detected in BM form B-ALL patients who relapsed or achieved CR. And use FISH and xenograft modeling to assess whether CD34^+^CD19^−^CD22^+^ cells initiate leukemogenesis.	CD22 preceded CD19 in normal B-cell development and CD34^+^CD19^−^CD22^+^progenitors underlie phenotypic escape after CD19-directed immunotherapies.	[100]
**CRS**									
scRNA-seq	B-ALL	CD44v6, CD19	Post-infusion CD45^+^immune cells	8 mice	Cell	6,511	scRNA-seq was performed on CD45^+^ cells at two periods (day 2/7 after fever) after CAR T-cell infusion.	Human circulating monocytes, rather than CAR T-cells, were primarily responsible for the systemic release of IL-6, which ultimately caused CRS.	[108]
Single-cell cytokine profiling, flow cytometry	NHL	CD19	IPs	20 patients	Human	500,000	Single-cell multiplex cytokine profiling was performed on the IPs of 20 NHL patients.	Higher product PSI was associated with clinical response and severe CRS while higher numbers of IL-17A-producing polyfunctional CAR T (Th17)-cells was associated with severe ICANS.	[58]
**ICANS**									
scRNA-seq	NA	CD19	Human brain, lung pericytes, PBMCs, mice brain cells	Mice and the BRAIN Initiative Cell Census Network	Cell	#	scRNA-seq was performed on brain cells of 4 healthy mice and verified the analysis results of human brain, peripheral blood and pulmonary parietal cells from a scRNA-seq database.	CD19 was expressed in human brain mural cells that are critical for BBB integrity and this cell population might contribute to the neurotoxicity of CD19-directed immunotherapy including CAR T-cells therapy.	[113]
scRNA-seq	BCL	CD19	IPs, post-infusion CAR T-cells	72 patients	Human	956,647	After analysis of the HHV-6 public data sets of the two studies, scRNA-seq was performed on the IPs and CAR T-cells of 72 patients at different time periods after infusion as well as on PBMCs of 1 patient with HHV-6B overexpression on day 7/14/21 after treatment.	Reactivated HHV-6 carried by CAR T- cells may enter the CNS through OX40 receptors on BBB endothelial cells, resulting in the development of HHV-6 encephalitis, which has similar symptoms and requires differential diagnosis from ICANS.	[114]
CyTOF, scRNA-seq, scTCR-seq, CITE-seq	LBCL	CD19	Post-infusion CAR T-cells	32 patients	Human	6,316	CyTOF was performed on CAR T-cells from 32 patients on day 7/21 after infusion, and CAR T-cells from 9 of them were analyzed by scRNA-seq, scTCR-seq, and CITE-seq.	CD4^+^Helios^+^ CAR T-cells on day 7 after infusion manifested hallmark features of Treg cells and were associated with progressive disease and less severe neurotoxicity.	[90]
**On-target, off-tumor effects**									
scRNA-seq, flow cytometry	NA	B-lineage- derived malignant cells, AML, and solid tumors related target antigens	Cells in normal tissues/organs	Healthy donors, public scRNA-seq datasets	Cell	#	Analyze 121 target antigen expression patterns of CAR T-cells in 18 tissues and organs derived from normal human samples, and then compare the expression levels of antigens in malignant cells and nonmalignant cells.	The expression patterns of 121 target antigens in normal tissues or organs were obtained at the single-cell level, which facilitated revealing the reason for on-target, off-tumor toxicity in special tissues/organs.	[116]
scRNA-seq	#	*	Cells from the human cell landscape and the adult human cell atlas	40 donors	Cell	427,118	Analyze the expression of 591 CAR targets in various cell types across different normal tissues from the public scRNA-seq databases.	A more stringent cutoff by defining a CAR target as a potentially risky gene had identified targets in the public databases that caused potential on-target, off-tumor toxicity.	[117]
**Engineered CAR T-cells**									
scRNA-seq, genome-wide CRISPR/Cas9 knockout screening	GBM	IL13Rα2	Activated and inactivated engineered and control CAR T-cells	Healthy donors	Cell	37,898	Genome-wide CRISPR/Cas9-based knockout screening was performed on CAR T-cells to identify essential regulators of effector activity. scRNA-seq was performed on engineered (knockout of TLE4 or IKZF2) and control CAR T-cells with or without stimulation by tumor cells.	CRISPR screening identified targets including TLE4 and IKZF2, knockout of which resulted in the preservation or expansion of certain CAR T-cell subsets displaying transcriptional signatures of superior effector function and inhibited exhaustion responses.	[130]
scRNA-seq, CRISPR/Cas9 genome editing system	NB	GD2	Activated and inactivated engineered CAR T-cells	2 healthy donors	Cell	79,317	scRNA-seq was performed on antigen-specific stimulated or unstimulated CAR T-cells.	The feasibility of preparing TRAC-targeted CAR T-cells by CRISPR/Cas9 technology and virus-free method was proved and TRAC-integrated CAR T-cells showed higher proportion of memory phenotype, less depletion phenotype and lower degree of differentiation.	[132]
scRNA-seq, CRISPR/Cas9 genome editing system	B-NHL	CD19	IPs, engineered CAR T-cells, PBMCs,	3 patients	Cell	63,789	scRNA-seq was performed on CAR T-cells from 3 NHL patients before CAR T-cell infusion and on day 7/12/28/29 after infusion.	The feasibility of preparing PD1-targeted CAR T-cells by CRISPR/Cas9 technology and virus-free method was proved and non-viral, PD1-integrated CAR-T cells exhibited enhanced antitumor ability.	[131]
scRNA-seq	MM, PAAD	MSLN, CD19	Post-infusion tumors, engineered CAR T-cells, non-naive CD8^+^ T cells	Mice	Mouse	NA	scRNA-seq was performed on tumors on day 7 after treatment with CAR T-cells.	CAR T-cells delivered RN7SL1 via extracellular vesicles, which was selective to immune cells in TME and could directly elicit favorable changes in myeloid/DC subsets that helped activate endogenous CD8^+^ T cells.	[133]
scRNA-seq	MM	TRP1	TILs untreated or treated with CAR T-cells	Mice	Mouse	9,767	scRNA-seq was performed on TILs of MM mice untreated or treated with CAR T-cells.	Superkine IL-2 and IL-33 expressing CAR T-cells exhibited a potent, universal antitumor response and shifted the TME from immune suppressive to immune stimulatory in the absence of preconditioning.	[134]
**Combination therapy**									
scRNA-seq	LCA	ROR1	IPs, pre-infusion and post-infusion lung tumors	Mice	Mouse	16,672	scRNA-seq was performed on IPs and lung tumors at three time points (untreated, 6 h after injection of Ox/Cy before CAR T-cell infusion and day 10 after CAR T-cell infusion).	The lymphodepletion regimen Ox/Cy activated lung tumor macrophages to produce multiple T-cell-recruiting chemokines that facilitated infiltration of CAR T-cells into tumors and remodel the immunosuppressive TME.	[120]
scRNA-seq, flow cytometry	BRCA	Her2	Post-infusion CD45^+^immune cells, CD4^+^CAR T-cells	Mice	Mouse	128,000	scRNA-seq was performed on CD45^+^ immune cells and CD4^+^ CAR T-cells isolated from TME on day 7/10 after Th/Tc17 CAR T -cell infusion with or without DMXAA.	DMXAA promoted CAR T-cell migration and persistence by generating a chemokine milieu that promoted CAR T-cell recruitment and modulating the immunosuppressive TME through alterations in the balance of immune-stimulatory and suppressive myeloid cells.	[123]
CITE-seq, RNA-seq, flow cytometry, ATAC-seq, scRNA-seq	CC, OV	Lewis Y	Untreated and pretreated T cells	Mice	Mouse	NA	CITE-seq, RNA-seq and ATAC-seq were performed on T cells from vehicle- or CDK4/6i-treated CC tumors. scRNA-seq was performed on in vitro–activated T cells and in vivo-derived T cells respectively. Animal models were used to explore the efficacy of CDK4/6i combined with CAR T-cells.	CDK4/6i-pretreated T cells exhibited increased memory phenotype and immune persistence. And combination of CDK4/6i and CAR T-cells in ovarian cancer mice significantly improved the effectiveness and persistence of tumor control.	[126]
**Locoregional delivery of CAR T-cells**									
CyTOF	B-ALL, NHL, DLBCL	CD19	IPs, PMBCs, BM, post-infusion CAR T-cells	3 patients	Human	NA	CyTOF was performed to analyze the trafficking and functional proteins expression in CAR T-cells across patients’ tissues, including leukapheresis T cells, IPs, CAR T-cells in peripheral blood, BM, and CSF post infusion.	CAR T-cell product showed increased expression of trafficking and activation molecules, and patients’ CAR T cells from peripheral blood, BM and CSF showed spatiotemporal alteration in trafficking, activation, maturation, and exhaustion expression, with distinct signature in the CSF niche.	[140]
scRNA-seq	CNSL	CD19	Post-infusion CAR T-cells	Mice	Mouse	NA	scRNA-seq was performed on CAR T -cells isolated from mice BM with both CNS and systemic lymphoma 68 days after ICV or IV CAR T-cell infusion.	Compared with IV, exposure of CAR T-cells to CSF after ICV infusion led to a metabolic reprogramming that favored the formation of memory and exhibited enhanced antilymphoma activity.	[138]
scRNA-seq	DIPG, DMG	GD2	IPs, post-infusion CSF cells	4 patients	Human	65,598	scRNA-seq was performed on IPs and CSF cells from patients after IV and ICV administration.	Transcriptomic analyses of IPs and CSF showed heterogeneity in response between participants and administration routes. Particularly, ICV administrations were associated with less immunosuppressive cell populations in CSF compared with IV infusions.	[141]
**CAR target selection**									
Machine learning, scRNA-seq, CITE-seq	#	#	Tumor, tumor-infiltrating normal and reference normal cells	9 patients, public scRNA-seq datasets	Cell	#	scRNA-seq, random forest, convolutional neural networks were performed on identification of targets for logical switch-based CAR therapy and CITE-seq was performed on transcriptome-coupled epitope mapping.	The large-scale tumor-normal single-cell meta-atlas were leveraged to select gene pairs ( AND, OR and NOT switch targets) that contributed most to discrimination between individual malignant and normal cells. And the results were validated in ovarian cancer and colorectal cancer.	[154]

*CAR* Chimeric antigen receptor, *scRNA-seq* single-cell RNA sequencing, *ALL* Acute lymphoblastic leukemia, *MHC* Major histocompatibility complex, *CITE-seq* Cellular indexing of transcriptomes and epitopes by sequencing, *scATAC-seq* single-cell assay for transposase-accessible chromatin sequencing, *MM* Multiple myeloma, MSLN Mesothelin, *BCMA* B-cell maturation antigen, *IPs* Infusion products, *BM* Bone marrow, *LV* Lentiviral vector, *VSV* Vesicular stomatitis virus, *BCL* B cell lymphoma, *T*_*H*_ Helper T cell, *Treg* Regulatory T, *TACI* Cyclophilin ligand interactor, *PBMCs* Peripheral blood mononuclear cells, *scTCR-seq* single-cell T-cell receptor sequencing, *TIGIT* T cell immunoglobulin and ITIM domain, *CLL* Chronic lymphocytic leukemia, *NHL* Non-Hodgkin’s lymphoma, *CyTOF* Cytometry by time-of-fligh, *PCL* Plasma cell leukemia, *RP* ribosomal protein, *TME* Tumor microenvironment, *GBM* Glioblastoma, *PAAD* Pancreatic adenocarcinoma, *CAE* continuous antigen exposure, *NK* Natural killer, *ChIP-seq* Chromatin immunoprecipitation sequencing, *Mm* Malignant melanoma, *LBCL* Large B-cell lymphoma, *PR* Partial response, *PD* Progressive disease, *CR* complete remission, *PFS* Progression-free survival, *DCs* Dendritic cells, *MCL* Mantle cell lymphoma, *CTLs* Cytotoxic T lymphocytes, *T*_*SCM*_ Stem cell-like memory T cell, *T*_*CM*_ Central memory T cell, *HSPCs* Hematopoietic stem/progenitor cells, *FBM* Fetal bone marrow, *FISH* Fluorescence in situ hybridization, *CRS* Cytokine release syndrome, *PSI* Polyfunctional strength index, *ICANS* Immune effector cell-associated neurotoxicity syndrome, *BBB* Blood brain barrier, *HHV* Human herpesvirus, *CNS* Central nervous system, *AML* Acute myeloid leukemia, *TLE4* Transcription factor transducin like enhancer of split 4, *IKZF2*198 Ikaros family zinc finger protein 2, *NB* Neuroblastoma, *TRAC* The T cell receptor alpha constant, *TILs* Tumor-infiltrating lymphocytes, *LCA* Lung cancer, *Ox* Oxaliplatin, *Cy* Cyclophosphamide, *BRCA* Breast cancer, *CC* Colon cancer, *OV* Ovarian cancer, *CDK4/6i* Cyclin-dependent kinases 4 and 6 inhibitor, *DLBCL* Diffuse large B-cell lymphoma, *CSF* Cerebrospinal fluid, *CNSL* Central nervous system leukemia, *ICV* intracerebroventricular, *IV* intravenous, *DIPG* Diffuse intrinsic pontine glioma, *DMG* Diffuse midline glioma * ATCC and Broad Institute’s Cancer Cell Line Encyclopedia, # details in citation

## Basics of CAR T-cells

**Fig. 1:**
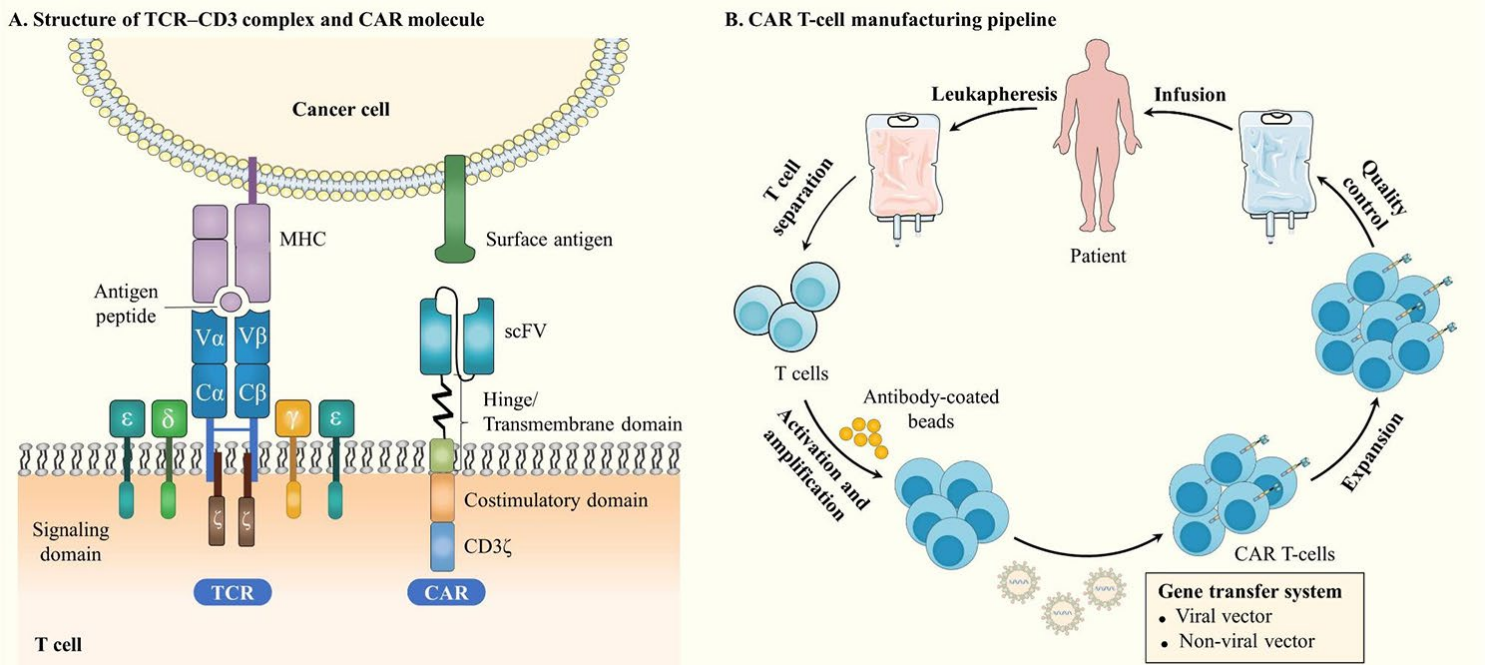
The basics of CAR T-cells. **(A)** Illustration of the basic structure of the conventional TCR-CD3 complex and CAR molecule. **(B)** Flow scheme of the CAR T-cell manufacturing process. Autologous CAR T-cell manufacturing typically begins with leukapheresis of the patient. Then, the T cells are activated and amplified with antibody-coated beads. After that, the CAR construct is introduced into the T cells, typically by viral or non-viral vectors. Finally, CAR T-cells are expanded to the required amount and then infused into the patient after quality control testing

CAR T-cell product heterogeneity is affected by CAR structure [24–26], T cell subtype [27] and product manufacturing process [28], which influences the efficacy and safety of CAR T-cell therapy. Single-cell sequencing technologies can systematically evaluate the impact of the above factors on the final CAR T-cell product, guiding the rational design and optimization of CAR T-cell therapy. CAR is an engineered receptor that is composed of three main parts: the extracellular, transmembrane and intracellular domains **(**Fig. 1A**)**. According to the differences in the design of intracellular domains and the adoption of cytokines and ligands, CAR molecules have been developed for five generations [29]. Different combinations of molecular modules of the CAR have different effects on the phenotype and function of CAR T-cells, such as the selection of the costimulatory domain [26] and the immunogenicity of the single-chain variable fragment (scFv) [24, 25]. For T cell subtypes, the CD4:CD8 ratio and the composition of different T cell subtypes affect the antitumor ability of CAR T-cell therapy. For instance, when the CD4:CD8 ratio is 1:1, the synergistic antitumor ability can have a better effect [30–32]. CAR T-cells with less-differentiated naive and early memory features are related to a higher rate of durable clinical remission [33, 34]. Exhausted T cells with higher expression of inhibitory immune checkpoint receptors are associated with poorer clinical outcomes [35]. In addition, the difference in CAR T-cell lineage clones based on the T cell receptor (TCR) is also an important factor leading to the heterogeneity of CAR T-cell products [36].

During the manufacturing process, every step may contribute to the heterogeneity of the final CAR T-cell product [28]. The traditional manufacturing process of CAR T-cells begins with the collection of peripheral blood mononuclear cells (PBMCs) from patients undergoing leukapheresis. Next, T cells that have been further enriched from PBMCs are stimulated with anti-CD3/anti-CD28 monoclonal antibodies to induce T cell activation, and then lentiviral vectors, gamma-retroviral vectors or other delivery methods are used to engineer CAR molecules into T cells. Subsequently, these generated CAR T-cells are expanded in vitro to reach the required amount of cells for either experimental testing or clinical treatment [37, 38] **(**Fig. 1B**)**. Moreover, T cells can also be extracted from healthy donors (HDs) to prepare allogeneic CAR T-cells [39].

## Single-cell sequencing technologies for CAR T-cells

Single-cell sequencing technologies used in CAR T-cell research mainly include scRNA-seq, single-cell T-cell receptor sequencing (scTCR-seq), single-cell assay for transposase-accessible chromatin sequencing (scATAC-seq), cytometry by time-of-flight (CyTOF), cellular indexing of transcriptomes and epitopes by sequencing (CITE-seq) and single-cell multiplexed secretome proteomics.

### scRNA-seq for CAR T-cell research

**Fig. 2:**
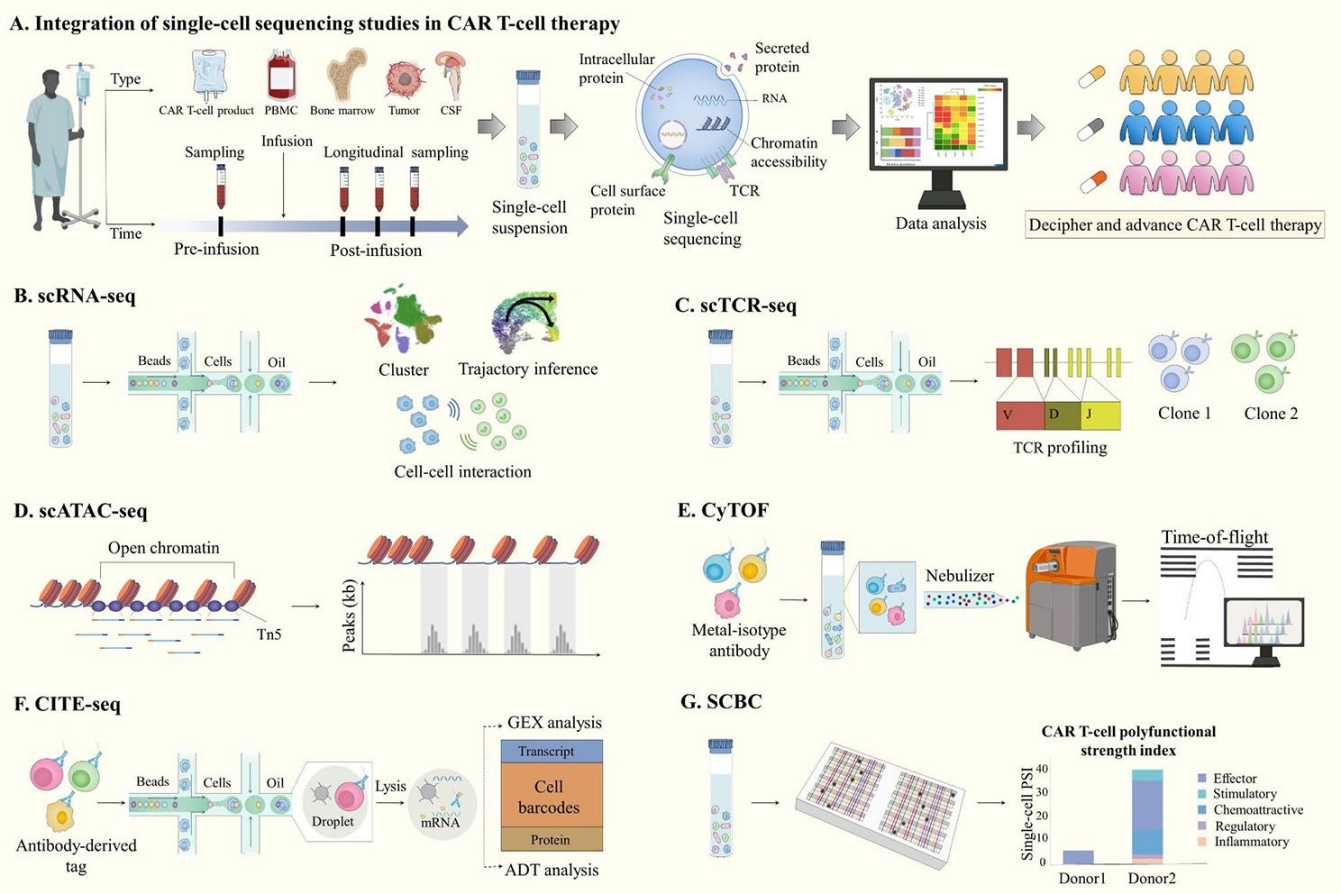
Single-cell sequencing technologies for CAR T-cell therapy. **(A)** Schematic overview of single-cell multi-omics analysis of CAR T-cell therapy. **(B)** scRNA-seq measures the transcriptome from the entire cell. **(C)** scTCR-seq measures the V(D)J sequence of TCR and gene expression profiles in tandem. **(D)** scATAC-seq identifies open regions of chromatin by inserting adapters with Tn5 transposase to map regions of transcription factor binding. **(E)** CyTOF analyzes a high-dimensional, multi-parametric quantification of protein detection with metal-isotype antibody, inductively coupled plasma ionization, and time-of-flight detector. **(F)** CITE-seq allows the simultaneous assessment of the transcriptome and surface or intracellular protein expression of individual cells immunostained with oligonucleotide-coupled antibodies. **(G)** SCBC measures multiple secreted proteins with arrays of microchambers that are decorated with ordered arrays of antibodies against target proteins

scRNA-seq is currently the most widely used single-cell sequencing technology. Every scRNA-seq experiment follows a similar basic strategy, including sample dissociation, single-cell capture, cell lysis, mRNA reverse transcription, cDNA amplification, library construction, high-throughput sequencing, and data analysis [40, 41] (Fig. 2A, B). The samples of CAR T-cell research mainly include CAR T-cell products, PBMCs, bone marrow (BM), cerebrospinal fluid (CSF) and tumor tissue. Adequate sample preparation is a prerequisite for generating reliable single-cell transcriptomics results. The general dissociation process includes tissue dissection, mechanical mincing, enzymatic/proteolytic extracellular matrix (ECM) breakdown and selective enrichment. Since CAR T-cell products and PBMCs are single-cell suspensions, the dissociation step is eliminated, avoiding the generation of stress genes in the process and retaining the proportion of various cell types, which is crucial for the reliability of single-cell sequencing data.

The droplet-based platform (10x Genomics BD) is the most commonly used scRNA-seq platform in the field of CAR T-cells. Using a microfluidic chamber, the droplet-based method can separate individual cells into an oil-based microdroplet. A single droplet contains gel microbeads with mRNA-capturing primers in conjunction with a unique molecular barcode alongside an enzyme/reagent mix, which is required for cell lysis and reverse transcription. Single cells in the droplets are lysed, followed by reverse transcription, cDNA amplification, and the generation of a barcoded sequencing library. Then, the samples are processed for sequencing. The obtained raw data require quality control, which is crucial to the subsequent exploration of biological questions from downstream analysis at the cell/gene level. Downstream analyses include simple quantification of gene expression to in-depth examination of cell heterogeneity, lineage transitions, cell-cell interactions, etc. Comprehensive tutorials of workflow and analysis of scRNA-seq have been established [40, 41].

### Other single-cell sequencing technologies

scTCR-seq sequences the 5′-end transcript and can simultaneously detect the V(D)J sequence and transcript of a single cell (Fig. 2C). It can not only characterize the clonotypic diversity of the CAR T-cell population but also correlate T cell clones with their functional phenotypes, such as activation, memory, and exhaustion, providing information on the role of CAR T-cell clonotypic phenotypes in driving the immune response [42–44]. By inserting sequencing adapters into accessible regions of the genome with Tn5 transposase, scATAC-seq allows the measurement of genome-wide open chromatin regions to identify both activated genes and cis-elements (such as promoters and enhancers), as well as to postulate the TFs to which cis-elements are bound [45] (Fig. 2D). At present, scATAC-seq is mainly used to characterize the epigenetics of CAR T-cell differentiation and exhaustion [46, 47]. CyTOF, also known as mass cytometry, is a technology that combines flow cytometry with mass spectrometry for multi-parameter analysis. By conjugating more than 100 antibodies with unique isotopically pure metals to overcome the overlaps in fluorophore spectrum, CyTOF could detect intracellular and extracellular protein expression accurately at the single-cell level [48, 49] (Fig. 2E). As a platform for multiplexed data analysis, CITE-seq utilizes an oligonucleotide-barcoded antibody conjugated to the cell surface antigen that can achieve parallel characterization of cell populations on the basis of the expression of surface protein markers and the transcriptome landscape [50] (Fig. 2F). Cytokines are potent secreted regulators that play an important role in CAR T-cell function [51, 52]. The single-cell barcode chip (SCBC) is one of the most advanced single-cell proteomic devices for measuring secreted cytokines [53]. SCBC uses concentrated arrangements of microfabricated compartments (typically < 1 nL volume) with spatially barcoded capture sites (currently commercialized by Isoplexis) for highly multiplexed single-cell analysis of up to 42 cytokines related to CAR T-cell function, such as effector, stimulatory, regulatory and inflammatory molecules [54] (Fig. 2G). Since CAR T-cells exhibit significant heterogeneity in cytokine secretion, polyfunctional CAR T-cells and the polyfunctionality strength index (PSI) are used to describe CAR T-cell subsets capable of co-producing multiple cytokines at the single-cell level, which has been utilized to predict the clinical outcome of patients [55–58].

## Deciphering and advancing CAR T-cell biology

### CAR T-cell product heterogeneity

For CAR structures, the selection of the costimulatory domain is a critical factor to explore. Although CAR T-cells with CD28 or 4-1BB have similar clinical efficacy, they differ in kinetics and phenotype [26]. scRNA-seq showed that they had distinct transcriptional expression profiles, whether in the baseline or activated state, which indirectly reflected the different transcriptional regulatory mechanisms [59–61]. In addition to verifying that 4-1BB CAR T-cells have the gene encoding the memory phenotype and longer persistence compared with CD28 CAR T-cells [59, 60], multiple studies have observed that 4-1BB CAR T-cells express more MHC II genes [59–61]. This is presumably beneficial for co-application with tumor vaccines to increase antigen presentation to enhance epitope spread, but increases the risk of host-graft rejection of allogeneic “off-the-shelf” CAR T-cells [59].

For T cells, allogeneic CAR T-cells derived from HDs are an important means to expand the clinical accessibility of CAR T-cell therapy [62–64], but the intrinsic heterogeneity, function and safety of HD-derived allogeneic CAR T-cells have not been systematically evaluated. A study combined scRNA-seq and CITE-seq to characterize the differences between HD and patient-derived CAR T-cells in transcriptome, phenotype, and metabolic characteristics and found that HD-derived CAR T-cells were at a higher level of activation [61]. Moreover, the upregulation of MHC II genes indicated that HD-derived CAR T-cells may have stronger and faster antitumor efficacy but increased the risk of being cleared by the host immune system. HD-derived CAR T-cells were also associated with lower granulocyte-macrophage colony-stimulating factor (GM-CSF) expression than patient-derived CAR T-cells [61], which is a stimulator of CRS [65, 66], consistent with the clinically observed lower incidence of CRS in allogeneic CAR T-cells [67].

The difference in manufacturing processes is a crucial factor leading to CAR T-cell product heterogeneity. Previous studies have observed that the selection of fresh or cryopreserved PBMCs as primary material led to discrepancies in the efficacy of CAR T-cell products [68, 69]. A recent single-cell study suggested that this might be related to the number of Treg cells, which are notoriously intolerant of freezing [70]. In addition, the efficiency of viral transduction of CAR molecules influences CAR T-cell fitness and antitumor efficacy. Different profiles of CAR T-cells expressed by different CAR molecules (CAR^High^, CAR^Low^) at the bulk and single-cell levels confirm that CAR^High^ T-cells have stronger tonic signaling, activation and exhaustion [71]. Furthermore, characterizing gene regulatory networks found that CAR^High^ T-cells were regulated by the exhaustion-related regulators RFX5, NR4A1, and MAF [71]. Notably, cells with low or even negative expression of CAR express interferon-induced transmembrane (IFITM) 2 and IFITM3, which prevent viral vector entry and are probably potential drug targets to overcome the inefficiency of CAR transduction [72]. For vector bias to CAR T-cell function, scRNA-seq revealed that the transduction of vesicular stomatitis virus (VSV)-lentiviral vectors (LV) promoted the transition of CAR T-cells to a central memory phenotype, while the transduction of CD8-LV promoted the transition of CAR T-cells to a cytotoxic phenotype [72].

### Antigen-specific stimulation of CAR T-cells

The activation mechanism of CAR T-cells is quite different from that of innate T cells. Compared with TCR-induced activation, the full molecular landscape of downstream signaling in CAR-induced activation remains elusive, which is partly due to the heterogeneity of CAR T-cell products. On the one hand, different CAR T-cell phenotypes differ in their ability to respond to antigens. On the other hand, not all cells in CAR T-cell products harbor CAR expression, which easily confuses the analysis of the cells upon antigen encounter. Several studies have used scRNA-seq and other single-cell sequencing technologies to resolve the heterogeneity of CAR T-cell products under different conditions of unstimulated, CAR-induced stimulated and TCR-induced stimulated CAR T-cells [59, 61, 73–75]. Due to the presence of ligand-independent tonic signaling, CAR T-cells in the unstimulated state are regulated by a mixture of early activation, exhaustion signatures, and cytotoxic activities [61]. After CAR-induced activation, CAR T-cells present highly mixed T_H_1/T_H_2 cell signaling. The levels of cytokines, such as IFN-γ, TNF-α, GM-CSF, IL-5 and IL-13, show great heterogeneity among different cell subsets [75]. Since GM-CSF is highly expressed in many CAR T-cells, GM-CSF^+^ CAR T-cells can be regarded as in a functionally active state, which is different from conventional T cells [61, 75]. Notably, CD4^+^ and CD8^+^ CAR T-cells showed high expression of cytotoxic cytokines, indicating that both cell types had killing functions [75]. Moreover, some CAR T-cell subsets upregulated the expression of the immune checkpoint genes CTLA4 and PD-1 and the immunosuppressive cytokine genes IL-10 and TGFB1 and downregulated the costimulator genes inducible costimulatory (ICOS) and OX40, which may be a mechanism to maintain immune homeostasis after activation [75]. In addition, some studies have also observed that some activated CAR T-cells did not change the transcriptomic profiles, and a few CAR T-cells showed signs of exhaustion in the early stages after activation [74, 76]. The relevant mechanism is still unclear, and it may be attributed to the CAR T-cell manufacturing process, tonic signaling, or cell-source specificity [74]. The gene expression was different between CAR T-cells stimulated by TCR and CAR-specific stimulation, with the former more specifically enriched in T cell activation genes (e.g., IFN-γ, IL-3, and CCL4) [59, 73].

### The dynamic performance of CAR T-cells in vivo

**Fig. 3:**
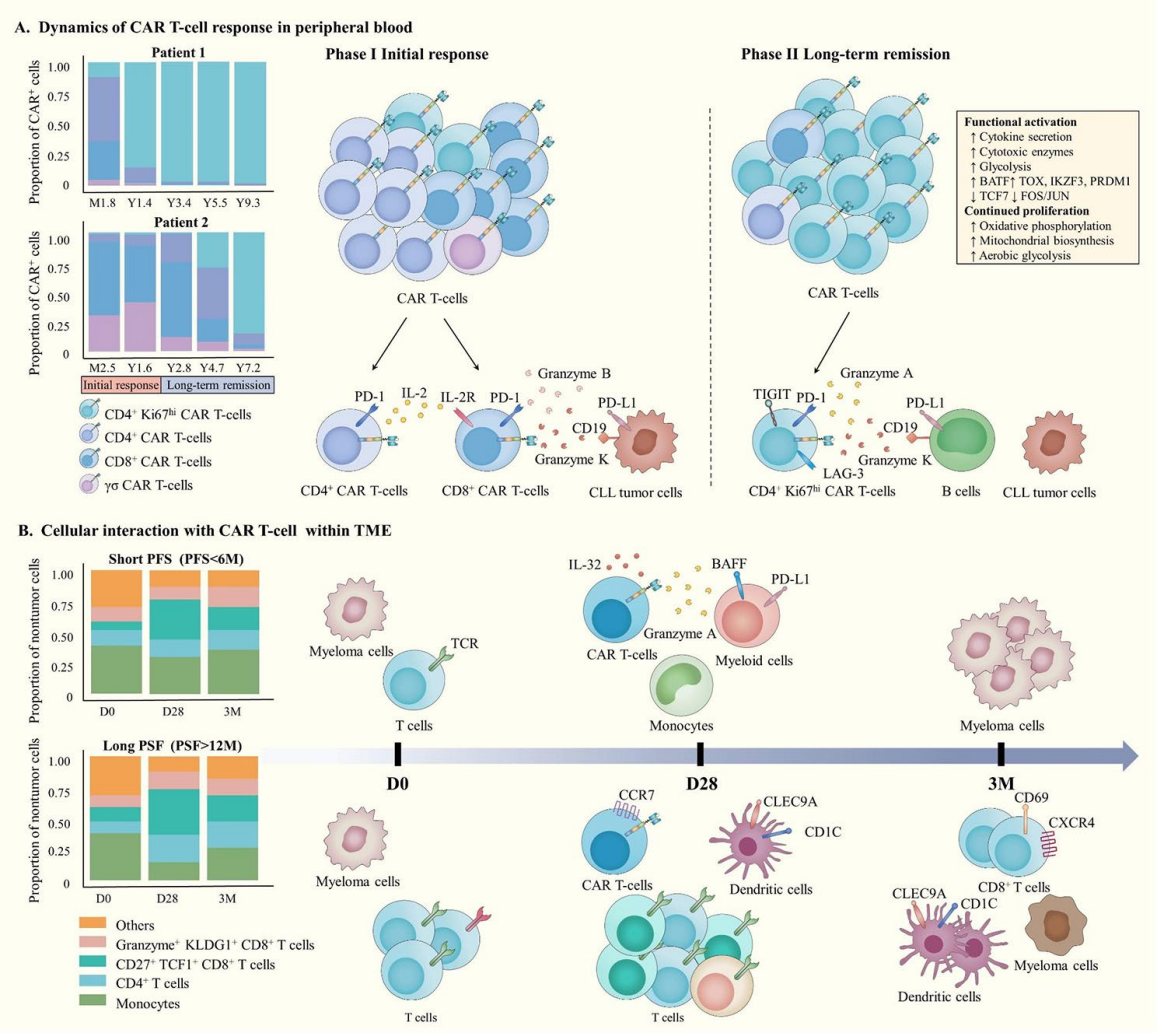
The dynamic biology of CAR T-cells in the peripheral blood and tumor microenvironment. **(A)** Schematic diagram of CAR T-cell dynamics in different phases in two CLL patients with clinical remission for more than 10 years after the infusion of CAR T-cells. (Left) The adjacent stacked bar plots exhibit the proportion of each CAR T-cell population at different time points. (Right) In the initial stage, CD4^+^ CAR T-cells provide support to CD8^+^ CAR T-cell responses via IL-2. In the long-term remission phase, the expression of the GZMA and GZMK genes was strongly upregulated, while GZMB was not highly expressed. The persistence of Ki67^hi^CD4^+^ CAR T-cells may partly be driven by healthy B cells. **(B)** Schematic diagram of cellular changes in bone marrow samples at different times in the TME (classified as pre-therapy, 28 days (D28) or 3 months (3M) following CAR T-cell treatment) of patients with short PFS (< 6 months) or long PFS (> 6 months). (Left) The adjacent stacked bar plots show the proportion of nontumor cells at different times. (Right) For patients with short PFS, there were terminal differentiation markers in bone marrow T cells at day 0 (D0). Monocytes/macrophages enriched with myeloid cells expressing BAFF and PD-L1 and CAR T-cells showed a more differentiated/effector phenotype at D28. At 3M, myeloma cells were increased and similar to the baseline phenotype. For patients with long PFS, the diversity of TCR was higher at D0 and increased at D28, accompanied by CAR T-cells with a more naive phenotype and enrichment of dendritic cells. CD8^+^ T cells at 3M had higher expression levels of genes associated with human bone marrow residence/retention, such as CXCR4 and CD69, and the myeloma cell phenotype was different from baseline

CAR T-cells after infusion will experience rapid expansion as well as differentiation and exhibit long-term persistence of atypical patterns in metabolism and clearance [77–79]. The dynamic performance of CAR T-cells varied in both initial response and long-term remission, reflecting the function of CAR T-cells and the interaction between CAR T-cells and the host. Heterogeneity of CAR T-cell products affects the differentiation of CAR T-cells to different cellular dynamics and cell fates. A study utilized scRNA-seq and scTCR-seq and revealed that early CAR T-cell proliferation in tisa-cel responders was characterized by expansion of memory-like CD8^+^ CAR T-cell clones that differentiated into IL7R^+^ effector memory CAR T-cells, while axi-cel responders exhibited more heterogeneous populations. Among them, CD8^+^ CAR T-cells had stronger upregulation of activation marker PDCD1 and the immune checkpoint regulator SLAMF6 [70]. However, even if the same CAR T-cell product was activated by the same antigen, different CAR T-cell subpopulations led to different patterns of expansion and displayed divergent differentiation trajectories [36, 76]. However, clusters with high expression of cytotoxicity and proliferation genes usually predominated the post-infusion CAR T-cell functional groups [36, 76]. In addition, a study using scRNA-seq and scTCR-seq found an effector precursor CD8^+^ CAR T-cell with a unique transcriptional profile TIGIT^+^CD27^−^CD62^Low^ in the initial infusion sample [76]. It was subsequently the main source of the majority of CAR T-cells with an effector phenotype in patients [76]. As tumor cells were cleared, most CAR T-cells at the remission phase further developed into long-lived memory cells and stayed in the “resting primed” state with minimal energy consumption to prevent relapse [36, 80]. These processes were conserved in the evolution of CAR T-cells targeting different antigens in different hematological malignancies [36, 76, 80]. Of note, in a recent study, two patients with CLL who achieved complete remission (CR) for up to 10 years had an initial response dominated by cytotoxic CD8^+^ CAR T-cells in their peripheral blood, followed by a long-term remission stage dominated by cytotoxic and proliferative Ki67^hi^CD4^+^ CAR T-cells [81] (Fig. 3A). The CD4^+^ CAR T-cells displayed a non-classical memory phenotype and a state of ongoing activation and proliferation. Meanwhile, they expressed cytotoxic genes, such as GZMA, GZMK and PRF1, as well as genes related to oxidative phosphorylation pathways. In vitro culture showed that the long-persisting CD4^+^ CAR T-cells were capable of killing CD19-expressing target cells directly. Nevertheless, in other studies, almost no CD4^+^ CAR T-cells were observed in patients in remission, and cytotoxic effects were absent [80]. Thus, the importance of CD4^+^ CAR T-cells in long-term disease control should be considered in conjunction with patient-specific characteristics, and the universality of the gene expression profiles needs to be further validated and explored in large-scale clinical cohort.

### Cellular interactions with CAR T-cells

The function of CAR T-cells is closely associated with endogenous immune cells in the circulation system and tumor cells, immune cells and stromal cells in the TME. The complex cellular interactions involved in the function of CAR T-cells are critical to understanding the mechanism of action of CAR T-cells [82]. A study performed scRNA-seq on CAR T-cell products and PBMCs from plasma cell leukemia (PCL) patients after receiving CAR T-cell treatment. The ligand-receptor analysis showed the extensive interaction between proliferating CAR T-cells and cytotoxic CAR T-cells, as well as between CAR T-cells and endogenous T cells, and the gene ontology (GO) analysis found it associated with T cell activation, cell-cell adhesion, and TNF-related pathways, which indicated that CAR T-cells may establish a new immune environment by recruiting endogenous T cells [80]. Hence, CAR T-cells act as not only killers but also regulators. In fact, CAR T-cells can also reshape the TME and activate both innate and adaptive immunity to produce synergistic antitumor immunity by releasing IFN-γ [83, 84]. It was observed in two studies that IFN-γ derived from CAR T-cells promoted a more activated and less suppressive TME in two different animal models (B-cell lymphoma and glioblastoma), with concurrent activation and an increase of T lymphocytes and natural killer (NK) cells and the upregulation of myeloid cells expressing more antigen processing and presentation-related genes. Subsequent functional experiments confirmed that a lack of IFN-γ impaired the activation of host immune cells, which then affected the killing efficacy of CAR T-cells in vivo [85, 86].

CAR T-cells in continuous contact with tumor cells could easily lead to CAR T-cell exhaustion. A study performed bulk and single-cell sequencing of CAR T-cells continuously co-cultured with tumor cells in vitro (0, 20 and 28 days) and found that exhausted CD8^+^ CAR T-cells changed to a NK-like phenotype at the transcriptional, epigenetic and protein levels [87]. Meanwhile, gene expression levels of exhaustion markers and the NK signature increased under chronic antigen stimulation. Moreover, the transcriptional regulators ID3 and SOX4 were specifically expressed in exhausted NK-like CAR T-cell clusters, and their knockdown restored CAR T-cell potency. However, it is noteworthy that NK-like CAR T-cell clusters are not pre-existing but rather that CD8^+^ CAR T-cells acquire NK receptors via plasticity during prolonged antigen exposure. The causality of the transition and CAR T-cell dysfunction remains uncertain. Another study delineated the landscape for comprehensive and dynamic chromatin accessibility of CAR T-cells derived from an in vitro tumor cell co-culture system (0, 6 and 48 hours) via scATAC-seq [47]. Two subsets of exhausted CAR T-cells were defined. One is intermediate exhausted CAR T-cells with enriched motifs of transcription factors such as JUN, FOS, NFKB1, and BACH2, and the other is terminal exhausted CAR T-cells with enriched motifs of BATF, IRF4, and PRDM1. Similar changes in chromatin accessibility and transcriptional regulation patterns of exhausted CAR T-cells were also observed in patients. An in vitro experiment confirmed that CAR T-cells with reduced BATF and IRF4 expression exhibited better persistence and enhanced killing ability.

## Deciphering and advancing the efficacy and safety of CAR T-cell therapy

### Primary resistance

**Fig. 4:**
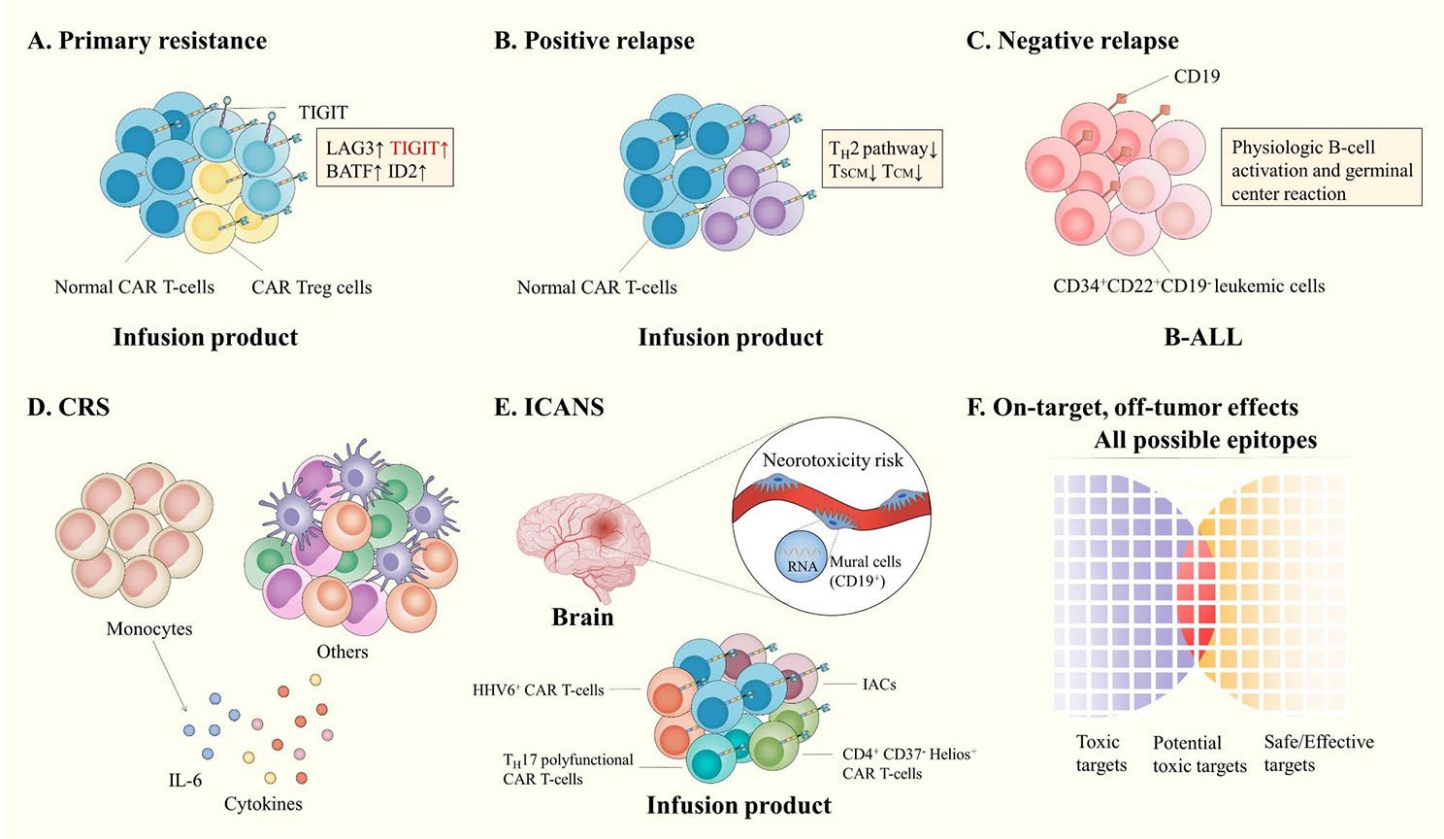
Mechanisms of resistance and toxicities. **(A)** Genes encoding immune checkpoints, such as lymphocyte activation 3 (LAG3) and T cell immunoglobulin and ITIM domain (TIGIT), as well as exhaustion-associated transcription factors, including basic leucine zipper ATF-like transcription factor (BATF) and inhibitor of DNA binding 2 (ID2), were highly expressed in infusion products. Among them, TIGIT has been used as a potential target with suggested beneficial efficacy in both in vitro and in vivo experiments. In addition, the number of CAR Treg cells also increased significantly in infusion products. **(B)** The T_H_2 pathway was absent, and early memory-like T cell subsets (T_SCM_ and T_CM_) were decreased in infusion products. **(C)** scRNAseq profiling revealed that CD19-negative leukemic cells were present before CAR T-cell therapy. CD34^+^CD22^+^CD19^−^ progenitor cells are B-ALL cells with negative CD19 expression that carry oncogenic lesions and lead to leukemia. In addition, regulatory programs of B-cell activation and germinal center reaction occurring in B-ALL cells can reduce CD19 expression. **(D)** IL-6, recognized as pivotal for CRS pathogenesis, is specifically and highly expressed by monocytes. **(E)** Targeting CD19^+^ mural cells, critical for BBB integrity, may lead to ICANS of CAR T-cell therapy. Reactivation of HHV-6^+^ CAR T-cells in the infusion product may cause ICANS. IACs, more IL-17 A polyfunctional T cells, and fewer CD4^+^CD57^–^Helios^+^ CAR T-cells in the infusion product are associated with high-grade ICANS. **(F)** Integrative analyses of single-cell sequencing datasets promote a deeper understanding of CAR antigen expression and identification of safer and more effective targets

Primary resistance refers to the inability to induce CR after CAR T-cell infusion. A study performed a whole-transcriptome scRNA-seq analysis of CD19 CAR T-cell products from 24 large B-cell lymphoma (LBCL) patients and found that exhausted CD4^+^ and CD8^+^ T cells were enriched within infusion products (IPs) of patients with partial response/progressive disease (PR/PD) 3 months after infusion. Moreover, the lymphocyte activation 3 (LAG-3) and TIGIT genes and the basic leucine zipper ATF-like transcription factor (BATF), inhibitor of DNA binding 2 (ID2) and other failure-related transcription factors were highly expressed. Nevertheless, patients achieving CR had more memory CD8^+^ T cells [88]. Other scRNA-seq data obtained from relapsed/refractory B-cell lymphoma patients before and after CAR T-cell infusion suggested that the upregulation of the exhaustion marker TIGIT in CAR T-cells was highly correlated with a lower clinical response. Moreover, subsequent experiments both in vitro and in vivo confirmed that blocking TIGIT could delay tumor progression and restore the antitumor function of CAR T-cells. In the future, immune checkpoint inhibitors against TIGIT may be the key to improving the clinical response in patients [89]. In addition, CAR Treg cells are also involved in primary resistance [70, 90]. Another scRNA-seq study deciphered IPs and PBMCs derived from LBCL patients before and after CAR T-cell infusion and found that the increasing number of CAR Treg cells in IPs was positively correlated with the nonresponse rate of patients. In vitro and in vivo models showed that CAR Treg cells inhibited the antitumor activity of CAR T-cells and led to tumor relapse in mouse models [70]. Similarly, a study used CyTOF to examine PBMCs from LBCL patients after CAR T-cell infusion and found that higher frequencies of cytotoxic CD4^+^ and CD8^+^ subsets of CD57^+^ T-BET^+^ CAR T-cells were associated with patients achieving CR, whereas higher numbers of CD4^+^ HELIOS^+^ CAR T-cells with a Treg cell phenotype were associated with disease progression. Furthermore, a logistic regression model that combined the percentage of CAR Treg cells and the level of lactate dehydrogenase (LDH) was a powerful predictor of durable complete response versus progression [90] (Fig. 4A).

Tumor cells can also induce primary resistance. Unbiased genome-wide loss-of-function screens in B-ALL and B lymphoma cell lines have revealed that tumor cells with inherent dysregulation of death receptor signaling could be resistant to CAR T-cell killing [91, 92]. Meanwhile, reduced expression of death receptor genes (FADD, BID, CASP8, and TNFRSF10B) was associated with patients with no response [91]. scRNA-seq results confirmed that the CAR T-cells from these patients expressed much higher levels of exhaustion markers [91].

### Relapse

Relapse, also called acquired resistance, refers to the phenomenon in which patients who initially respond to CAR T-cell therapy experience disease recurrence over time. The TME plays a considerable role in promoting tumor relapse because of the expansion of the failure phenotype and immunosuppression-related cells [93–95]. A study leveraged multi-level single-cell sequencing technologies to analyze BM samples from multiple myeloma (MM) patients after B-cell maturation antigen (BCMA) CAR T-cell treatment and analyzed its association with the length of progression-free survival (PFS) in the responders. They found that patients with a long PFS were usually associated with the increased appearance of CLEC9A^+^ dendritic cells, CD27^+^TCF1^+^ T cells and T cells expressing marrow-residence genes, while the expansion of KLRG^+^HLA-DR^+^ terminally differentiated T cells and the appearance of the immunosuppressed BAFF^+^PD-L1^+^ myeloid cells were related to a short PFS. In addition, the residual myeloma cells tended to have less-differentiated and stem-like characteristics, with upregulated epithelial-mesenchymal transition (EMT) genes, which may be related to tumor relapse [96] (Fig. 3B). Another scRNA-seq study analyzed tumor tissue from glioblastoma (GBM) patients before and after CAR T-cell therapy and revealed that phenotype remodeling occurred in recurrent GBM after CAR T-cell therapy with a transition from a mesenchymal-like (MES) phenotype to a mixed MES-like, neural progenitor-like (NPC) and astrocyte-like (AC) phenotype, suggesting that the coexistence of cells characterized by different GBM subtypes undermined the efficacy of CAR T-cell therapy [97]. Similarly, a scRNAseq study analyzed PBMC and BM samples before and after CAR T-cell treatment in patients with mantle cell lymphoma (MCL) and discovered that the number of endogenous cytotoxic lymphocytes (CTLs) was significantly decreased after relapse, while the expression of the immune checkpoint TIGIT was high, which impaired their killing ability. TIGIT was also highly expressed on MCL cells. Cell-cell interaction showed that the interaction between TIGIT on MCL cells and its ligand CD155 on CD16^+^ monocytes significantly increased after relapse, which might attenuate the ability of myeloid cells to present tumor antigens and lead to weaker antitumor immune surveillance. In conclusion, the acquired expression of TIGIT in CTLs and MCL cells may be a central mechanism resulting in relapse in MCL patients [98]. In addition, further studies have focused on the distinct mechanisms of relapse by investigating positive and negative relapse.

#### Positive relapse

Positive relapse refers to tumor relapse with targeted tumor antigen expression, generally caused by limited activation and expansion, as well as poor antitumor potency and persistence of CAR T-cells in vivo [93, 94]. Single-cell sequencing technologies have not only validated the previously identified mechanisms but also helped to discover some entirely new mechanisms of positive relapse (Fig. 4B). Bai et al. [73] analyzed the IPs from some ALL patients with CR and others undergoing positive relapse via scRNA-seq and found that a deficiency of T_H_2 function in CAR T-cells was associated with positive relapse patients. Cytokine module analysis indicated that T_H_2 cytokine modules were enriched in CR patients and absent in positive relapse patients. Differential expression analysis also showed that except for the T_H_2 pathway and T_H_2-related genes, including IL-4, IL-5, IL-13 and the upstream regulator GATA3, there was no significant difference in other immune programs, such as effector, activation, inflammation and chemokine modules, between CR and positive relapse patients. Therefore, the maintenance of T_H_2 function may be indispensable for the long-term remission of patients receiving CAR T-cell therapy. In addition to immune function, CITE-seq analysis revealed that differentiated subsets of T cells could also make a difference. Early memory-like T cell subsets T_SCM_ and T_CM_ were significantly decreased in both resting and activated CAR T-cells from positive relapse patients. Furthermore, a prognostic model including three biological indicators, CAR^+^T_H_2^+^ frequency, T_CM_ frequency, and T_EM_ + T_EF_ frequency, endowed good discrimination of patients with positive relapse and long-term remission, which may act as a biomarker for the prediction of risk and clinical response of patients.

#### Negative relapse

Negative relapse refers to tumor relapse without targeted tumor antigen expression [93]. The recognized mechanisms include the selection of pre-existing antigen-negative tumor cells, mutation, splicing variation, lineage switching-mediated target antigen loss and other factors affecting the presentation and expression of target antigens [99]. Several single-cell sequencing studies have validated the natural selection theory of the presence of antigen-negative tumor subclones before CAR T-cell treatment [100, 101] and unveiled a new mechanism for mediating negative relapse [102] (Fig. 4C). A study performed scRNA-seq on CD10^pos^CD19^pos^ cells and CD10^pos^CD19^neg^ cells of BM from patients with B-ALL before and after CAR T-cell treatment [101] and found that the gene expression profiles of the CD19^neg^ B-ALL cells detected before CAR T-cell treatment were similar to those of other B-ALL cells both before and after CAR T-cell treatment but were significantly different from those of other CD19^neg^ cells, such as NK cells and myeloid cells. Moreover, CaSpER and Ballele frequency (BAF) analysis showed that the potential pre-existing CD19^neg^ B-ALL cells harbored genomic deletions in the same chromosome location as the other B-ALL cells, further confirming the presence of true B-ALL cells that did not express CD19 before CAR T-cell treatment [101]. Another study further characterized antigen-negative expressing cells. The researcher applied bulk RNA-seq, scRNA-seq and flow cytometry to describe the gene expression of CD34^+^ hematopoietic stem cell progenitor (HSCP) development across healthy fetal, neonatal, and postnatal samples and found that CD22 was expressed before CD19, which is highly expressed in PreProB and ProB progenitors. CD34^+^CD19^−^CD22^+^ early/immature progenitors were prevalent in patients with relapsed B-ALL and increased the probability of relapse in patients receiving CD19-targeted therapy. Fluorescence in situ hybridization (FISH) showed that CD34^+^CD19^−^CD22^+^ early/immature progenitors in B-ALL patients carried an oncogene lesion, and subsequent animal testing demonstrated their ability to initiate leukemogenesis [100]. Since the initial number of antigen-negative expressing cells is quite small, it is usually difficult to detect by flow cytometry [101]. However, scRNA-seq can unambiguously detect these scarce antigen-negative subclones and thus can serve as an effective strategy for CAR T-cell selection before cellular product infusion. Regarding novel mechanisms leading to negative relapse, Im et al. used live cell imaging and CD19-specific antibody fluorescence and found that when CD19 CAR T-cells interacted with B-ALL cells, the CD19 surface protein of surviving B-ALL cells accumulated and internalized into cells and subsequently downregulated CD19 expression on the surface. This process is similar to the activation of normal B cells [103]. scRNA-seq and scATAC-seq of surviving leukemic cells co-cultured with CAR T-cells showed that the leukemic cell subsets with the lowest CD19 expression were significantly enriched for gene expression and regulators correlated with B-cell activation signatures and germinal center reaction. Bruton’s tyrosine kinase (BTK) inhibitors can inhibit this process and enhance CAR T-cell killing ability [102].

### Adverse events

#### CRS and ICANS

Cytokine release syndrome (CRS) is the most common and severe toxicity of CAR T-cell therapy [104–108]. CRS is generally considered to be a systemic inflammatory response induced by endothelial cell dysfunction, abnormal activation of macrophages, and the release of supraphysiological levels of various proinflammatory cytokines [106–109]. Although IL-6 is recognized as critical for CRS [104–108], the main cellular source releasing it remains unknown. An in vitro cytokine release assay of cultured CAR T-cells found that IL-6 secretion was closely related to monocytes, and the presence of IL-6 in monocytes was confirmed by intracellular staining results. scRNA-seq performed on CD45^+^ leukocytes isolated from humanized mice that developed CRS after CAR T-cell infusion confirmed that monocytes were the only cell population that consistently and specifically expressed high levels of IL-6. Overall, circulating monocytes, but not CAR T-cells, were major sources of IL-6 during CRS [108] (Fig. 4D). Moreover, single-cell cytokine profiling revealed that higher product PSI was associated with grade ≥ 3 CRS [58]. Compared to patients with grade 0-1 CRS, patients with grade ≥ 2 CRS had a higher PSI of CD4^+^ CAR T-cells, especially PSI of IL-8 and MCP-1 [57], which are involved in the recruitment of neutrophils and monocytes/macrophages [110, 111]. Moreover, PSI combined with CAR T-cell expansion or pretreatment serum IL-15 levels was more indicative of severe CRS [58].

Immune effector cell-associated neurotoxicity syndrome (ICANS) is another common toxicity related to CAR T-cell therapy. Activation of endothelial cells in the brain during inflammation and the destruction of the integrity of the blood brain barrier (BBB) may play a key role in the occurrence and development of ICANS [104, 105, 112]. Single-cell sequencing technologies elucidated the pathogenesis of ICANS (Fig. 4E). By analyzing human brain scRNA-seq data, Parker et al. discovered a rare population of cells that co-expressed CD19 and CD248, the cell marker of mural cells. Specific expression of CD19 in mural cells was confirmed by exclusion of B-cell confounding and immunohistochemistry of several regions of the human brain. This described a possible mechanism for ICANS, namely, CD19 CAR T-cells targeting mural cells, thus increasing BBB permeability and causing circulating inflammatory cytokines and CAR T-cells to enter the central nervous system (CNS) [113]. These findings may caution the use of intrathecal administration of CD19 CAR T-cells for primary CNS lymphoma. Another scRNA-seq study found that reactivated human herpesvirus 6 (HHV-6) carried by CAR T-cells may enter the CNS through OX40 receptors on BBB endothelial cells, resulting in the development of HHV-6 encephalitis, which has similar symptoms and requires differential diagnosis from ICANS [114]. Moreover, single-cell sequencing technologies could also identify some biomarkers from CAR T-cell products that predict an increased risk of severe ICANS, such as ICANS-associated cells (IACs) with a monocyte-like transcriptional signature [88], an increased number of polyfunctional CAR T-cells producing IL-17A [58], and low levels of CD4^+^Helios^+^ CAR T-cells [90].

#### On-target, off-tumor effects

On-target, off-tumor effects refer to CAR T cell-mediated recognition and lysis of non-malignant tissues expressing the target antigens because the antigens recognized by CAR T-cells are mostly tumor-associated antigens (TAAs) expressed in both normal and malignant cells [115]. The development of single-cell sequencing technologies has improved the resolution of target antigen analysis, providing valuable guidance on on-target, off-tumor effects evaluation (Fig. 4F). By integrating publicly available scRNA-seq databases, the antigen expression profiles of CAR targets were delineated in normal as well as malignant tissues, highlighting the importance of identifying differential expression levels of target antigens in patients before CAR T-cell therapy [116]. To further search for potential off-target CAR antigens, Jing et al. analyzed two single-cell databases, the human cell landscape (HCL) and adult human cell atlas (AHCA), and defined the CAR targets measurable in more than 100 nonimmune cells and more than 2% of the total cells in at least three normal tissues as a potentially risky gene (PRG) [117]. By employing this criterion, they identified PRGs neglected by bulk expression analysis, such as EGFR, PSCA and KDR (VEGFR2), and suggested that close surveillance was needed for early signs of off-target toxicity [117]. Of note, Parker et al. discovered that even 0.15% (12/7906) CD19-measurable human brain mural cells could lead to ICANS [113], but whether such a low level of expression had toxic impact on other tissues remained unknown. Moreover, different physiological and disease states may also affect the expression of target antigens to some extent. Therefore, a comprehensive atlas of human antigen expression is vital for understanding the on-target, off-target effects, and follow-up experiments may be needed to confirm the off-target toxicity.

## Deciphering and advancing strategies for CAR T-cell therapy

### Combination therapy

Combining CAR T-cells with other therapies, including chemotherapy, radiotherapy, hematopoietic stem cell transplantation, and other immunotherapies, is regarded as a promising strategy to overcome challenges and enhance the effectiveness of CAR T-cell therapy [118, 119]. In a study, cyclophosphamide (Cy) and oxaliplatin (Ox) were used innovatively to replace the conventional chemotherapy regimen Cy and fludarabine (Flu) prior to CAR T-cell infusion [120], which has been shown to promote T cell infiltration into tumors [121]. scRNA-seq profiles of murine lung tumors treated with Ox/Cy and CAR T-cells revealed the expression of T-cell-recruiting chemokine genes, including CXCL16 and CCL5, in multiple cell types in the TME, such as macrophages and DCs, which facilitated early infiltration of CAR T-cells into tumors partially through CXCR6 and CCR5. Subsequently, IFN-γ produced by tumor-infiltrating CAR T-cells led to the recruitment and activation of iNOS^+^ tumor macrophages. As the main source of chemokines, iNOS^+^ macrophages upregulate the expression of the CXCR3 ligands CXCL9 and CXCL10, binding to CAR T-cells expressing CXCR3 to facilitate their further infiltration and therefore initiating a positive feedback loop supporting CAR T-cell recruitment to tumors [120]. A patient receiving this combination regimen also achieved improved clinical responses [120]. Moreover, stimulator of IFN genes (STING) pathway activation enhanced T cell recruitment and effector cell function in tumors [122]. Another scRNA-seq study combined the STING agonists DMXAA/cGAMP with CAR T-cells to treat mice with breast cancer. The results demonstrated that compared to CAR T-cell infusion alone, DMXAA/cGAMP could regulate the inhibitory TME by increasing proinflammatory myeloid cells, reducing myeloid-derived suppressive cells and increasing the expression of chemokines that facilitated CAR T-cell recruitment and persistence at the tumor site. In addition, it could also promote the conversion of CAR T-cells to the proinflammatory phenotype to improve antitumor effects [123]. Furthermore, cyclin-dependent kinase 4 and 6 inhibitors (CDK4/6i) are widely used in cancer therapy [124]. In addition to inhibiting tumor proliferation by blocking the cell cycle [125], they were also found to promote the long-term antitumor effects of endogenous T cells through immunomodulatory effects [126]. A study combining scRNA-seq and CITE-seq found that T cells in the spleen of mice pretreated with CDK4/6i upregulated effector and memory-related genes, suggesting that CDK4/6i might enhance the cytotoxic effects of T cells and promote the differentiation of memory subsets that maintain long-term antitumor immunity. The combination of CDK4/6i and CAR T-cells in ovarian cancer mice significantly improved the effectiveness and persistence of tumor control [126].

### Engineered CAR T-cells

Designing innovative engineered CAR T-cells to optimize CAR T-cell therapy is an important area to be explored [127–129]. Single-cell sequencing technologies can help to systematically evaluate the features of engineered CAR T-cells. A study using genome-wide clustered regularly interspaced short palindromic repeats (CRISPR) screening discovered that Ikaros family zinc finger protein 2 (IKZF2) and transducin-like enhancer of split 4 (TLE4) may be linked with the functional suppression and exhaustion of T cells, thus affecting the killing effects of CAR T-cells on GBM stem cells. scRNA-seq performed on IKZF2-KO (knockout) and TLE4-KO CAR T-cells revealed that these cells displayed enhanced cytotoxicity and immune stimulation transcriptional signatures while prohibiting exhaustion, indicating their superior effector function against tumor cells [130]. In addition, two pilot studies used CRISPR-Cas9 gene editing technology for CAR gene transduction instead of traditional viral transduction [131, 132]. scRNA-seq found that CAR T-cells integrating the CAR gene at the T-cell receptor α constant (TRAC) locus and the PD1 gene locus both expressed a memory-like phenotype and less exhaustion-associated transcriptional signatures, which was related to higher potency [131, 132]. Obviously, the site-directed integration of CAR genes by CRISPR may be superior to random insertion by viral vector transduction.

In addition, many studies have attempted to modify engineered CAR T-cells to induce endogenous antitumor immunity and thus reshape the TME. One study designed a novel CAR T-cell (RN7SL1 CAR T-cell) that secreted non-coding RNA RN7SL1 in the form of extracellular vesicles (EVs), which would be preferentially taken up by immune cells to enhance endogenous antitumor immunity. scRNA-seq performed on tumor tissues of mice infused with RN7SL1 CAR T-cells showed that RN7SL1 could reduce suppressive myeloid cell subsets, increase inflammatory DCs expressing costimulatory genes, and activate amplification of effector-memory endogenous CD8^+^ T cells. Supported by these features, RN7SL1 CAR T-cells could perform an effective killing function even in a poorly immunogenic tumor [133]. Similarly, scRNA-seq suggested that CAR T-cells overexpressing superkine IL-2 (Super2) and IL-33 (Super2^+^ IL-33 CAR T-cells) could induce the conversion of M2-like macrophages to M1-like macrophages that highly express antigen presentation genes in TME, upregulate the ratio between CD8^+^ effector T cells and immunosuppressive Tregs, recruit and activate endogenous innate and adaptive immune cells, including tumor-specific T cells. The increased antitumor efficacy of Super2^+^ IL-33 CAR T-cells was observed in a variety of animal models [134].

### Locoregional delivery of CAR T-cells

CAR T-cells are usually administered intravenously to treat hematological malignancies, but for solid tumors, locoregional and intratumoral CAR T-cell delivery are also included [13, 135]. In the treatment of CNS tumors, intracerebroventricular and intrathecal administration of CAR T-cells has already shown positive outcomes in both preclinical and early clinical trials [135–137], which is probably related to the different microenvironments of CSF compared to PBMCs [7, 138, 139]. Two studies applying scRNA-seq and CyTOF respectively showed that CAR T-cells exposed to CSF can promote the formation of a memory-like phenotype through metabolic reprogramming, resulting in higher antitumor activity [138], and increase the expression of activation markers and trafficking/homing signatures, which may facilitate the migration of CAR T-cells to the CNS [140]. In the clinical study by Majzner et al. [141], glioma patients received a regional intracerebroventricular administration after the first GD2 CAR T-cell infusion through the vein. In addition to further radiographic and clinical benefits, local administration was associated with less systemic toxicity, such as CRS. Moreover, scRNA-seq found that intracerebroventricular administration reduced the number of myeloid cells with immunosuppressive properties compared with intravenous administration. In the future, by analyzing the distinct properties of CAR T-cells in PBMCs and CSF or monitoring the regional T cell dynamics more closely related to the TME, single-cell sequencing technologies may provide more insights into the treatment of CNS tumors.

## Perspectives for CAR target selection

An ideal CAR T-cell target has the following advantages: (1) coverage: expression on the vast majority of tumor cells; (2) specificity: tumor-specific expression and minimal expression in healthy tissues; (3) stability: continuous and stable expression to avoid antigen escape [142]. However, identifying suitable CAR T-cell targets has been challenging, especially for solid tumors, due to the co-expression of antigens between tumor cells and non-malignant cells, as well as the highly heterogeneous expression among tumor cells. Integrating single-cell multi-dimensional omics data is a promising strategy for CAR target selection.

Previous studies have integrated transcriptomics and/or proteomics data from analyses of both malignant and non-malignant tissues for target discovery [143–146]. Bosse et al. compared bulk RNA-seq results of neuroblastomas and normal tissues and identified Glypican 2 (GPC2) as a potential CAR T-cell target antigen, which has significant differential expression and extracellular epitopes [146]. However, bulk gene or protein expression data reflect average differences across tissues. Single-cell sequencing technologies with higher resolution can facilitate accurate identification of differentially expressed markers of tumor cell subsets and even rare but important tumor subsets, including cancer stem cells. Furthermore, homogenous target antigen expression can be assessed based on the fraction of tumor cells expressing each antigen. However, the sequencing depth of single-cell sequencing is still insufficient, and the average number of detected genes in a single cell is approximately 2000, with a massive amount of genes missing. With the improvement of sequencing depth, single-cell sequencing technologies may become a powerful tool for CAR target selection.

After screening targets that are specifically overexpressed in tumor cells, the specificity of targets should be verified via multi-organ single-cell atlases, such as HCL [147] and human cell atlas (HCA) [148]. A data portal (CARTSC) integrates HCL and HCA to visualize the expression of CAR target expression in normal tissues at the single-cell level [117]. At present, since most of the single-cell atlases of human organs have been completed and are available as open resources, the single-cell data of specific tissues can be directly obtained for expression verification of CAR targets [149–153]. Based on integrating published scRNA-seq data of tumor and normal tissues/organs, two recent studies systematically analyzed the expression of CAR T-cell target antigens and captured the rare cell types that had previously been omitted in the evaluation of bulk tissues [116, 154]. Particularly, through a machine-learning-based algorithm and the index ECF (expressing cell fraction), Kwon et al. selected ideal gene pairs for dual-target CAR T-cells that were controlled by logical switches (that is, AND, OR and NOT), promoting further tumor coverage and specificity [154].

The selected CAR T-cell target antigens require further validation of target expression on the surface of the cell membrane through proteomics techniques (such as flow cytometry). In addition, it is necessary to evaluate the target antigens in terms of function, immunogenicity, clinical response and other aspects. In particular, CAR T-cell products with novel targets need to be carefully evaluated in rigorous preclinical studies and early clinical trials.

## Conclusions and future perspectives

Although CAR T-cell therapy has achieved tremendous clinical success in hematological malignancies, challenges including high manufacturing costs, disease relapse, and adverse events remain to be overcome, and its efficacy in solid tumors requires urgent improvement. The application of single-cell sequencing technologies is a prospect to address these challenges. Single-cell sequencing technologies have natural advantages in deciphering CAR T-cell therapy since CAR T-cells are cellular products and single-cell suspensions. Currently, single-cell sequencing technologies mainly serve as evaluation platforms for deciphering the biological characteristics of CAR T-cell products and the therapeutic response of patients after CAR T-cell infusion. When identifying crucial cell populations and molecular features in certain pathophysiological processes, they can also provide potential strategies to advance CAR-T cell therapy. In addition, single-cell sequencing technologies have the potential to screen ideal CAR targets with sufficient coverage, high specificity and stable expression. Comprehensive single-cell atlases of normal and diseased tissues can be an effective tool for understanding off-target effects and validating candidate targets. It is worth noting that some limitations still exist in single-cell sequencing technologies [155], such as insufficient sequencing coverage and depth, sequencing bias, and high overall cost. Batch effect and data integration across experiments and different sequencing platforms are areas of particular attention that require continuous optimization and standardization of the data processing and analytical pipelines. To meet the demands of clinical accessibility, the accuracy, repeatability, stability and reliability of sequencing data also need to be continuously optimized. Standardized protocols for data analysis as well as physician-friendly interfaces and software should be developed for clinicians. We anticipate that the research benefits brought by single-cell sequencing technologies will push the limits of technical development and accelerate the standardization of computational analytical methods.

**Fig. 5:**
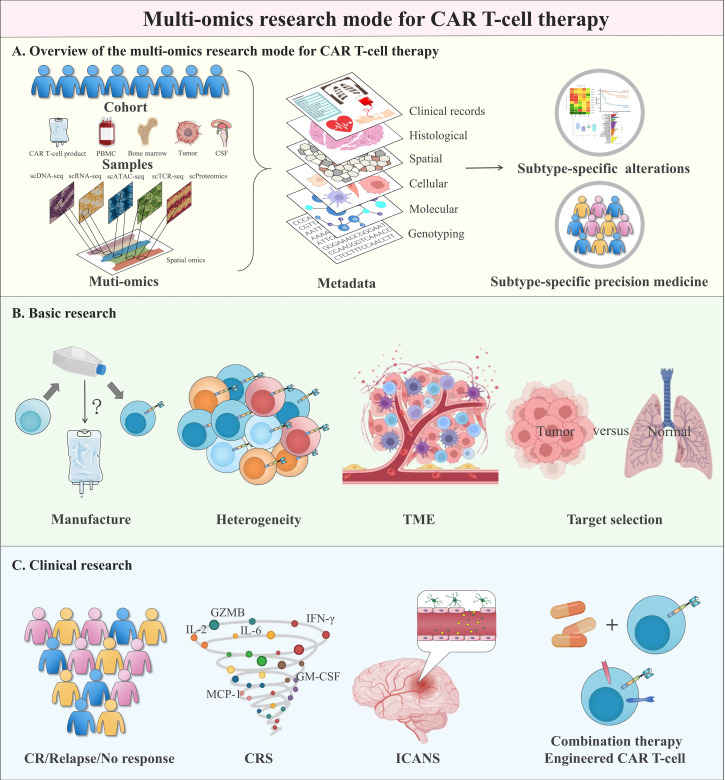
The multi-omics research mode for CAR T-cell therapy. **(A)** In the multi-omics research mode,  a large clinical cohort with complete clinical information as well as biological samples and single-cell multi-omics technologies would be combined to create multi-level metadata from micro to macros, which provides guidance for the development of precision medicine. In the field of CAR T-cell therapy, integration of multi-dimensional omics data promotes a comprehensive description of the molecular regulatory network during CAR T-cell functional processes, leading to the discovery of new mechanisms and targeted treatment strategies for CAR T-cell therapy and thus bringing more insights into this field at both the basic and clinical levels. **(B)** Basic research is dedicated to illustrating the basic biological characteristics of CAR T-cells, including the effect of each manufacturing stage on the final cellular products, the heterogeneity in the evolution of functional phenotypes during the CAR T-cell functional process, the interaction between CAR T-cells and various cells within the TME and the selection of suitable target antigens, which will advance the safety and efficacy of CAR T-cell therapy. **(C)** Clinical question-driven research focus on tracing the reasons for different clinical outcomes (including resistance, relapse, and toxicities) in patients treated with CAR T-cells. Grouping patients with different characteristics and comparing differences in metadata may bring more meaningful discoveries and guide further improvement of CAR T-cell therapy, such as combination therapy and engineered CAR T-cells

Based on the application and broad prospects of single-cell multi-omics technologies in the field of CAR T-cell therapy, we propose a multi-omics research mode to facilitate high-quality research and further clinical translation of CAR T-cell therapy. First, the establishment of a large clinical cohort and a complete sample library is conducive to achieving a subsequent refined experimental design, including the collection of paired samples at multiple time points before and after CAR T-cell treatment and comprehensive clinical and prognostic characteristics of patients, such as disease type, patient baseline characteristics, CAR T-cell product type, infusion dose, and clinical outcome [156]. Sample types include CAR T-cell products, PBMCs, BM, tumors, CSF and target organ tissues. Advanced single-cell multi-omics technologies can be used as a tool for research implementation, including single-cell genomics, epigenomics, transcriptomics, proteomics and spatial transcriptomics, enabling target discovery, mutual validation of the experimental results, and searching for upstream and downstream molecules and pathways in multiple dimensions (Fig. 5A). Based on the above premises and current progress, both basic and clinical research can be guided by multi-omics research mode. The former is dedicated to answering questions about the basic biological characteristics of CAR T-cells, such as the impact of the CAR structure, functional phenotype and manufacturing process on the final CAR T-cell product (Fig. 5B). Notably, from the perspective of cell types, analyzing the interaction among CAR T-cells, endogenous immune cells, stromal cells and tumor cells in the TME of BM and solid tumors would help to explore the mechanisms of tumor cell immune escape leading to relapse and to identify intervention targets. The latter is driven by clinical scientific questions, such as relapse, adverse events, and clinical efficacy, which are common in CAR T-cell therapy. Comparison groups based on clinical outcomes can be set up to identify key differential cell populations and differentially expressed genes to reveal potential mechanisms and therapeutic targets, and functional validation can be conducted in cells or animal models. Furthermore, research applying multi-omics technologies in a large-scale clinical cohort can also be conducted. Based on artificial intelligence, machine learning algorithms, and unsupervised and unbiased methods, detailed subgroup analysis of patients’ clinical information would be performed to determine risk prediction, prognosis stratification, therapeutic classification and personalized therapy (Fig. 5C).

In summary, leveraging single-cell sequencing technologies has brought new cellular and molecular insights to deciphering and advancing CAR T-cell therapy, with the potential to answer significant scientific questions related to the efficacy and safety of CAR T-cell therapy and is likely to revolutionize the methods of diagnosis and treatment and promote further progress in precision medicine.

## Data Availability

Not applicable.

## Abbreviations

BAF: Ballele frequency
B-ALL: B-cell acute lymphoblastic leukemia
BATF: Basic leucine zipper ATF-like transcription factor
BBB: Blood brain barrier
BCMA: B-cell maturation antigen
BM: Bone marrow
B-NHL: B-cell non-Hodgkin’s lymphoma
BTK: Bruton’s tyrosine kinase
CAR: Chimeric antigen receptor
CDK4i/6i: Cyclin-dependent kinases 4 and 6 inhibitors
CITE-seq: Cellular indexing of transcriptomes and epitopes by sequencing
CLL: Chronic lymphocytic leukemia
CNS: Central nervous system
CR: Complete response
CRS: Cytokine release syndrome
CSF: Cerebrospinal fluid
Cy: Cyclophosphamide
CyTOF: Cytometry by time-of-flight
ECM: Extracellular matrix
EMT: Epithelial-mesenchymal transition
EVs: Extracellular vesicles
FISH: Fluorescence in situ hybridization
Flu: Fludarabine
GBM: Glioblastoma
GM-CSF: Granulocyte-macrophage colony-stimulating factor
GO: Gene ontology
GPC2: Glypican 2
HCA: Human cell atlas
HCL: Human cell landscape
HHV-6: Human herpesvirus 6
HL: Hodgkin’s lymphoma
HSCP: Hematopoietic stem cell progenitor
IACs: ICANS-associated cells
ICANS: Immune effector cell-associated neurotoxicity syndrome
ID2: Inhibitor of DNA binding 2
ID3: Inhibitor of DNA binding 3
IFITM: Interferon-induced transmembrane
IKZF2: Ikaros family zinc finger protein 2
IPs: Infusion products
LAG-3: Lymphocyte activation 3
LBCL: Large B-cell lymphoma
LDH: Lactate dehydrogenase
LVs: Lentiviral vectors
MCL: Mantle cell lymphoma
MHC: Major histocompatibility complex
MM: Multiple myeloma
Ox: Oxaliplatin
PBMCs: Peripheral blood mononuclear cells
PCL: Plasma cell leukemia
PD: Progressive disease
PFS: Progression-free survival
PR: Partial response
PRG: Potentially risky gene
PSI: Polyfunctionality strength index
scATAC-seq: Single-cell assay for transposase-accessible chromatin sequencing
SCBC: Single-cell barcode chip
scFv: Single-chain variable fragment
scRNA-seq: Single-cell RNA sequencing
scTCR-seq: Single-cell T-cell receptor sequencing
STING: Stimulator of IFN genes
TAAs: Tumor-associated antigens
TCR: T cell receptor
TIGIT: T cell immunoglobulin and ITIM domain
TLE4: Transducin like enhancer of split 4
TME: Tumor microenvironment
TRAC: T-cell receptor α constant
VSV: Vesicular stomatitis virus
